# Identification and Structure–Activity Relationship of Intestinal Epithelial Barrier Function Protective Collagen Peptides from Alaska Pollock Skin

**DOI:** 10.3390/md17080450

**Published:** 2019-07-31

**Authors:** Wenkui Song, Qianru Chen, Ying Wang, Yan Han, Hongwei Zhang, Bo Li, Guangli Yu

**Affiliations:** 1 School of Medicine and Pharmacy and College of Food Science and Technology, Ocean University of China, Qingdao 266003, China; 2 School of Food Science and Technology, Qingdao Agricultural University, Qingdao 266109, China; 3 School of Sports Media and Information Technology, Shandong Sport University, Rizhao 276826, China; 4 Technical Center of Entry-Exit Inspection and Quarantine, Shandong Entry-Exit Inspection and Quarantine Bureau, Qingdao 266002, China; 5 Zhongshan School of Medicine, Sun Yat-sen University, Guangzhou 510080, China; 6 Laboratory for Marine Drugs and Bioproducts, Pilot National Laboratory for Marine Science and Technology (Qingdao), Qingdao 266237, China

**Keywords:** collagen peptide, intestinal epithelium, barrier function, intestinal health, structure–activity relationship

## Abstract

The effect of collagen peptides (CPs) in intestinal mucosal protection has been approved in both cell and animal models. However, its structure–activity relationship and efficient peptide sequences are unclear, which hinders the in-depth study of its action mechanism and relative nutraceuticals and pharmaceuticals development. In this work, size exclusion chromatography, cation-exchange chromatography, and RP-HPLC were used to separate Alaska pollock skin-derived collagen hydrolysates based on their molecular weight, charge property, and hydrophobicity. The intestinal epithelial barrier function (IEBF) protective effect of separated peptide fractions were evaluated by tumor necrosis factor (TNF)-α-induced Caco-2 cell model. Results indicated that lower molecular weight (500–1000 Da) and higher hydrophilicity of CPs were related to better IEBF protective effect. Two high-efficiency IEBF protective peptide sequences, GPSGPQGSR and GPSGLLGPK with the corresponding molecular weights of 841.41 Da and 824.38 Da, were subsequently identified by UPLC-QToF-MS/MS. Their IEBF protective ability are comparable or even better than the currently used intestinal health supplements glutamine and arginine. The present findings suggested that the hydrophilic CPs, with molecular weight between 500 Da to 1000 Da, should be preferred in IEBF protective peptides preparation. GPSGPQGSR and GPSGLLGPK might have the potential of being IEBF protective ingredients used in intestinal health supplements and drugs.

## 1. Introduction

The intestine is a specialized organ in which dynamic interactions between host cells and the complex environment occur in addition to food digestion and absorption [1]. As the first physical barrier, the intestinal epithelial cell layer performs a vital role in against external factors and maintaining intestinal homeostasis, together with the commensal bacteria, the chemical barrier of the mucosal layer, and the cellular immune system [2]. The term “intestinal epithelial barrier function (IEBF)” is used here to refer to the ability of intestinal epithelial cells as a protective barrier that restrict noxious molecules permeation form intestinal lumen to the underlying tissues, while supporting nutrients and water transport. Compromised IEBF has been associated with a number of pathological conditions, both intestinal and systemic, such as inflammatory bowel diseases (IBDs), metabolic disorders, systemic inflammatory response syndrome (SIRS), etc. [3,4,5]. IEBF may therefore be a prognostic marker for disease pathophysiology; similarly, targeting the IEBF holds promise for therapy and for the prevention of diseases [2].

Current studies on nutrient supplementation have suggested that the functional dietary peptides derived from daily foods, such as casein-derived peptides, collagen-derived peptides, whey protein hydrolysates, eggshell membrane hydrolysates, gliadin peptide, and phosphopeptides (PPPs) from hen egg yolk phosvitin, have the potential to maintain, reinforce, or repair the intestinal mucosa dysfunction through mechanisms of action including modulating intestinal immune reactions, diminishing oxidative stress, or modifying IEBF by regulating the expression and distribution of tight junction proteins [6,7,8,9,10]. The potential for food-derived peptides to modify intestinal mucosa function and to contribute to disease treatment has been proved by plenty of preclinical studies [7,8]. However, to precisely define the mode of action and to promote the development of intestinal mucosa protective peptide products, the responsible peptide sequences need to be isolated. Nowadays, the information about the intestinal mucosa protective peptide sequences are limited. A casein peptide, Asn-Pro-Trp-Asp-Gln, was reported to inhibited allergen permeation partly by enforcing the tight junction of intestinal epithelial cells [11,12]. A buffalo milk-derived peptide (MBCP) was informed with therapeutic potential in IBD by helping to restore the intestinal epithelium integrity damaged by inflammation [13]. The structure–activity relationship of IEBF protective peptides has not been described yet.

Collagen peptides have been increasingly used as dietary supplement or active ingredient in nutraceuticals and functional foods development because of their broad spectra of physiological and pharmacological properties [14,15]. Marine-derived collagen peptides received more attention in the recent past due to their favorable characteristics and less consumer reservations compared to mammal-derived ingredients [16]. We have previously discovered that Alaska pollock skin-derived collagen peptides supplementation could reverse intestinal mucosa dysfunction and correspondingly diminish intestinal and systemic inflammation based on its protective effects on intestinal epithelial tight junction integrity in burned mice model [17]. In addition, collagen peptides were demonstrated to protect the intestinal epithelial barrier function in both burned mice and tumor necrosis factor (TNF)-α stimulated Caco-2 cell monolayers via enhancing intercellular tight junction, which is a primary structure in maintaining the IEBF, by regulating the expression and distribution of tight junction protein ZO-1 and occludin through NFκB and MAPKs-mediated myosin light chain kinase (MLCK) pathway [17,18,19,20].

The biological properties of peptides are influenced by certain structural features such as molecular weight, charge, basicity, hydrophobicity, and spatial conformation, which are determined by the primary sequence [21]. Herein, we plan to separate the Alaska pollock skin-derived collagen peptides according to their molecular weight, charge property, and hydrophobicity, to explore the structure–activity relationship of IEBF protective peptides and to identify the high-active IEBF collagen peptide sequences, which may provide the possibility for further studies about the specific action mechanism of IEBF protective peptide and offer the theocratical support on corresponding drug or functional foods development.

## 2. Results and Discussion

According to previous studies, TNF-α stimulated Caco-2 cell monolayers was employed in this study as an intestinal epithelial barrier dysfunction model [18,22,23]. Trans-epithelial electrical resistance (TEER) and 4 kDa fluorescein isothiocyanate-conjugated dextran (4 kDa FITC-dextran, FD-4) permeability are two common parameters to indicate the integrity and permeability of cell monolayers, which can be used to appraise the protective effect of collagen peptides on IBEF [24]. TEER is indicative of the ionic conductance of the paracellular channel in the epithelial monolayer and FD-4 permeability reflects the paracellular permeability to nonionic macromolecules and indicates the pore size of the tight junctions [25].

### 2.1. Effect of Molecular Weight on IEBF Protection of Collagen Peptides

**Figure 1:**
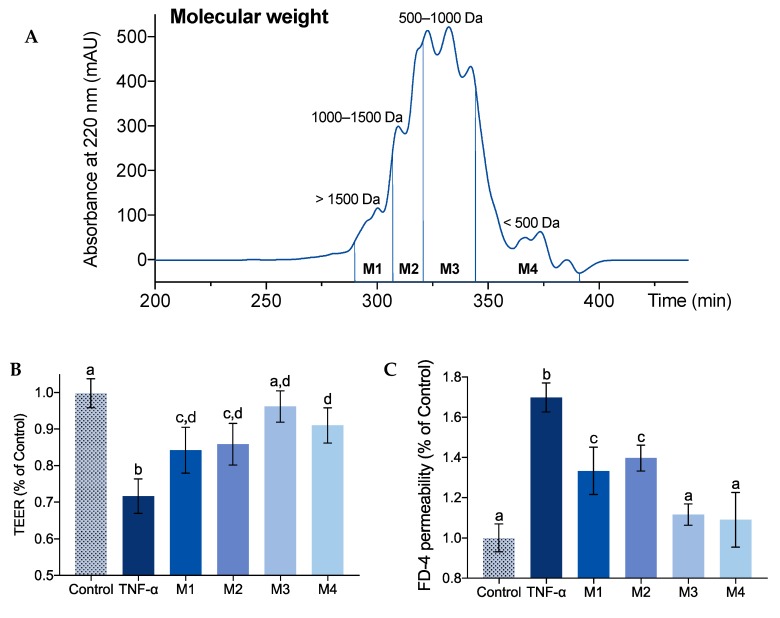
Chromatogram of collagen hydrolysates eluted by Sephadex G25 based on their molecular weight (**A**). Effect of separated fractions (M1 to M4) at the concentration of 1 mg/mL on trans-epithelial electrical resistance (TEER) (**B**) and fluorescein isothiocyanate-conjugated dextran 4 kDa (FD-4) permeability (**C**) of tumor necrosis factor (TNF)-α-induced Caco-2 cell monolayers. Values are expressed as mean ± SD, n = 6. a, b and c Means without sharing the same letter differ denote statistically significant differences (*p* < 0.05).

Alaska pollock skin (APS)-derived collagen was hydrolyzed by trypsin and sequentially separated into four fractions (Figure 1A): M1 (MW > 1500 Da), M2 (1000 Da < MW < 1500 Da), M3 (500 Da < MW < 1000 Da), and M4 (MW < 500 Da) by Sephadex G25 column, and their IEBF protective activities were determined. The results of TEER (Figure 1B) showed that all collagen peptide fractions could reverse TNF-α-induced decrease of TEER by 43.7–69.0% (*p* < 0.05 vs. TNF-α group). Among them, M3 presented the highest conversion of TEER at 86.0%, whose TEER is not significantly different from the control group (*p* > 0.05). Similarly, as shown in Figure 1C, all collagen peptide fractions showed obvious inhibition on FD-4 permeability increase by 52.3–87.1%, as compared with TNF-α group (*p* < 0.05). In contrast with M1 and M2, M3 and M4 presented more effective reduction of FD-4 permeability at 83.8% and 87.1%, respectively, which is similar to the FD-4 permeability level of control group (*p* > 0.05). Taken together, the relative abundant collagen peptide fraction M3 behaved best in protecting Caco-2 cells barrier function and, thus, was selected for the next step.

Molecular weight has been suggested to be a crucial factor affecting the bioactivities of protein hydrolysates [26]. The wide bioactivity spectrum of collagen peptides, such as anti-hypertensive, anti-oxidant, anti-microbial, and anti-photoaging activities, were associated with the contribution of low molecular weight peptides. Khiari et al. had reported that the Angiotensin-converting enzyme (ACE)-inhibitory and antithrombotic activities of mackerel skin gelatin hydrolysates were mainly due to the presence of low-molecular-weight peptides of 337 Da and 423 Da [27]. The efficient anti-oxidative collagen peptides derived from fish processing by-products were reported with the molecular weight below 1000 Da [28,29]. The collagen hydrolysates from pacific cod skin with the average molecular weight of 1200 Da exhibited effectively protect against UV irradiation-induced skin photoaging [30]. Here, combined with our previous findings [18], the lower molecular size of collagen peptides brings better IEBF protective effects, in general. The fraction with the molecular weight between 500 Da and 1000 Da demonstrates the most effective IEBF protection in TNF-α-induced Caco-2 cell model.

### 2.2. Effect of Charge Property on IEBF Protection of Collagen Peptides

**Figure 2:**
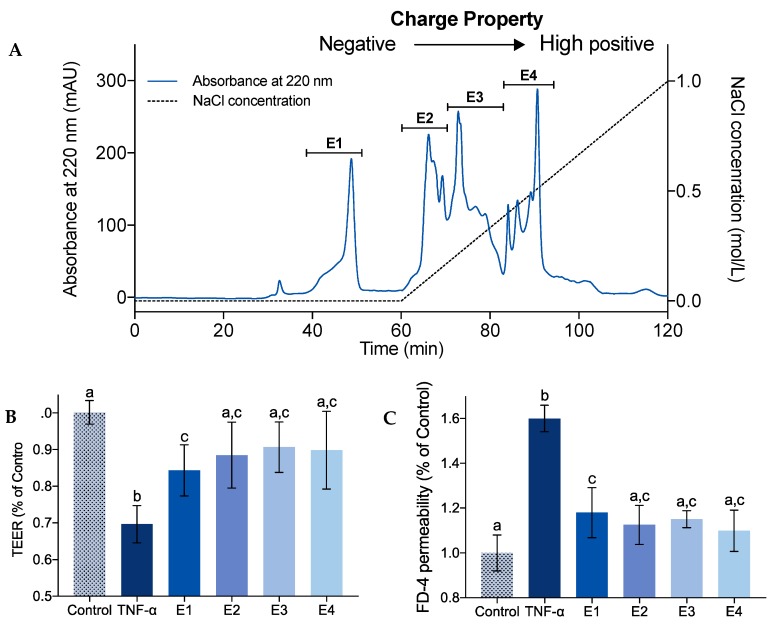
Chromatogram of collagen peptide fraction M3 (500 Da < MW < 1000 Da) eluted by a cation exchange column IexCap SP 6FF based on their charge property (**A**). Effect of separated fractions (E1 to E4) at the concentration of 1 mg/mL on TEER (**B**) and FD-4 permeability (**C**) of TNF-α-induced Caco-2 cell monolayers. Values are expressed as mean ± SD, n = 6. a, b and c Means without sharing the same letter differ denote statistically significant differences (*p* < 0.05).

The most effective collagen peptide fraction M3 from the former step was further fractioned into E1 to E4 by a cation exchange column IexCap SP 6FF based on their charge property (Figure 2A). E1 eluted by 0.02 M acetate buffer (pH 4.0) was a negatively charged fraction. E2 to E4 eluted by acetate buffer plus isocratic gradient NaCl, were positively charged fractions with accordingly increased charge. As shown in Figure 2B,C, all collagen peptide fractions could convert TNF-α-induced TEER drop and FD-4 permeability rise by 48.9–66.7% and 69.8–83.5%, separately, *p* < 0.05 vs. TNF-α group. No significant difference was found between these four fractions. It is notable that, after treatments, the cell condition in E2 to E4 group were recovered similarly to control group (*p* > 0.05). Because E4 had a relatively high content of collagen peptides among these four groups, it was chosen for subsequent experiments.

The charged residues of peptides play crucial roles in the ligand binding, which has provided an impetus for characterizing peptide structural features and biological activities [31]. A separation step of collagen hydrolysates based on their charge property is often performed for bioactive peptides identification [32]. However, the relationship between the charge property and bioactivities of collagen peptides is rarely mentioned. A series of studies had showed that the charge property of casein peptides affected their transepithelial transport, bioavailability, inhibition ability of LDL oxidation, and cyto-protection against H_2_O_2_-induced oxidative stress [33,34,35]. Meanwhile, in this study, only a tendency was observed that positively charged collagen peptides had better IEBF protective effect compared with negative charged ones, and no statistically significant difference was observed. It is indicated that charge property is not the main influencing factor of collage peptides in IEBF protection. 

### 2.3. Effect of Hydrophobicity on IEBF Protection of Collagen Peptides

**Figure 3:**
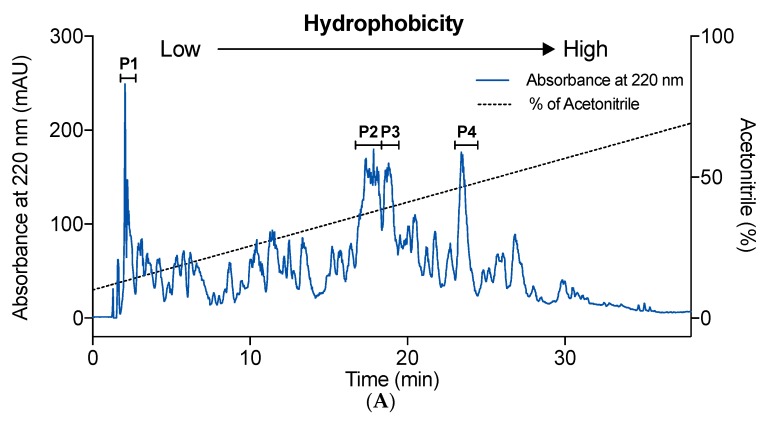

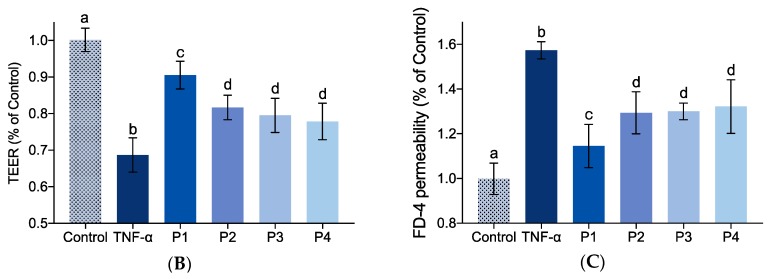
Chromatogram of collagen peptide fraction E4 eluted by a semi-preparatory Zorbax SB-C18 column based on their charge property (**A**). Effect of separated fractions (P1 to P4) at the concentration of 1 mg/mL on TEER (**B**) and FD-4 permeability (**C**) of TNF-α-induced Caco-2 cell monolayers. Values are expressed as mean ± SD, n = 6. a, b and c Means without sharing the same letter differ denote statistically significant differences (*p* < 0.05).

Collagen peptide fraction E4 was separated by a semi-preparatory Zorbax SB-C18 column, which allowed obtaining peptides fractions with different hydrophobic behaviors (Figure 3A). All of the chromatographic peaks were collected. The IEBF protective effect of four peaks, P1 to P4, with the absorbance at 220 nm over 100 mAU were assessed. All tested fractions enabled obvious reversion of TNF-α-induced TEER and FD-4 permeability changes in Caco-2 cell monolayers. Among those fractions, P1 exhibited the most potent IEBF protective activity, with a TEER recovery rate of 70.2% (Figure 3B, *p* < 0.05 vs. TNF-α group) and a FD-4 permeability inhibitory rate of 74.6% (Figure 3C, *p* < 0.05 vs. TNF-α group), which were significant better than other three fractions (*p* < 0.05 vs. P2–P4). No significant difference was observed among P2, P3, and P4. Then, the fraction P1 was further purified by UPLC-QToF-MS/MS.

Hydrophobicity, usually related to the content of hydrophobic amino acids, is commonly recognized to be an important factor of peptides that determines their interaction with several physiological targets and their bioactivities [36]. Peptides rich in hydrophobic amino acids (such as Ala, Leu, Val, Gly, and Pro) are well known to be related to the excellent anti-oxidant, anti-hypertensive, and anti-microbial properties, and can be a positive factor for accessibility to hydrophobic targets [37,38]. Hydrophilic peptides have been associated with prebiotic effect [39]. A short peptide, Asn-Pro-Trp-Asp-Gln, rich in hydrophilic amino acids, was reported to enhance intestinal barrier function [11]. The relationship of hydrophobicity and IEBF protective effect of peptides is first investigated in this study. Results prompted that the hydrophilic peptides fraction revealed the better IEBF protective action than hydrophobic ones in TNF-α-induced Caco-2 cell model.

### 2.4. Identification of High-Active IEBF Protective Collagen Peptide Sequences

**Figure 4:**
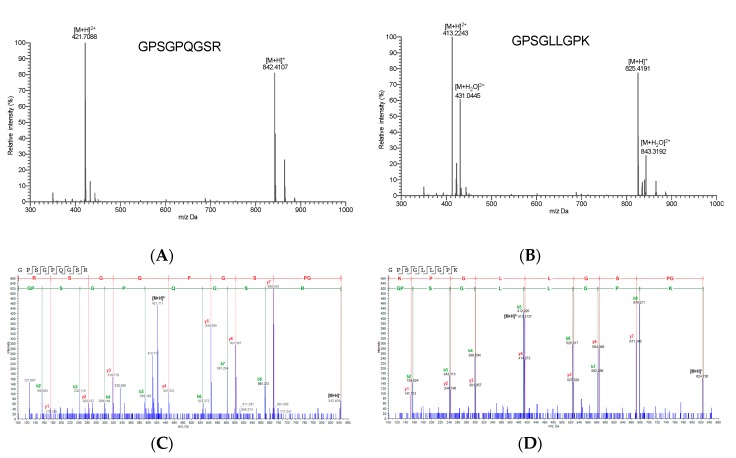
Mass spectrogram (MS) and corresponding secondary mass spectrogram (MS/MS) of two identified collagen peptide sequences from high-active intestinal epithelial barrier function protective collagen peptide fraction. (**A**) MS of Gly-Pro-Ser-Gly-Pro-Gln-Gly-Ser-Arg (GPSGPQGSR). (**B**) MS of Gly-Pro-Ser-Gly-Leu-Leu-Gly-Pro-Lys (GPSGLLGPK). (**C**) MS/MS of GPSGPQGSR. (**D**) MS/MS of GPSGLLGPK.

In order to clearly illustrated the active mechanism of IEBF protective peptides, the exact sequences need to be identified. As listed in Table 1, two collagen peptide sequences from P1 were identified to be Gly-Pro-Ser-Gly-Pro-Gln-Gly-Ser-Arg (GPSGPQGSR) and Gly-Pro-Ser-Gly-Leu-Leu-Gly-Pro-Lys (GPSGLLGPK) by UPLC-QToF-MS/MS. The measured molecular weight of GPSGPQGSR (Figure 4A) and GPSGLLGPK (Figure 4B) were 841.41 Da ([M+H]^+^ 842.41 Da) and 824.38 Da ([M+H]^+^ 825.67 Da), which agreed well with their theoretical masses of 841.40 Da and 824.48 Da. Their MS/MS spectrum are revealed in Figure 4C,D. These two sequences are novel peptides with IEBF protective effect from Alaska pollock, which have not been previously reported.

In line with the general feature of collagen peptide sequences, Gly-Pro-x and Gly-x-y are the most repetitive sequences that appeared in the identified two peptides. The C-terminus of these two sequences are both hydrophilic residues. Hydrophobic amino acids are the main components, contributing 56% and 78% of the composition, respectively, which is different from the other reported intestinal barrier function modulating peptide Asn-Pro-Trp-Asp-Gln [11].

### 2.5. Effect of Identified Collagen Peptide Sequences on IEBF of TNF-α-Induced Caco-2 Cell Monolayers

**Figure 5:**
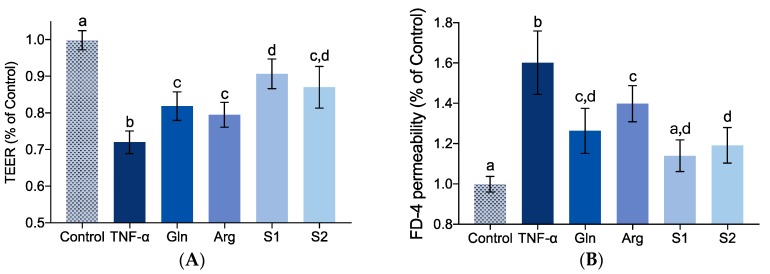
Effect of identified high-active IEBF protective collagen peptide sequences GPSGPQGSR (S1) and GPSGLLGPK (S2) at the concentration of 1 mg/mL on TEER (**A**) and FD-4 permeability (**B**) of TNF-α-induced Caco-2 cell monolayers. Glutamine (Gln) and Arginine (Arg) were used as positive contrasts. Values are expressed as mean ± SD, n = 6. a, b and c Means without sharing the same letter differ denote statistically significant differences (*p* < 0.05).

The effects of synthesized collagen peptide sequences GPSGPQGSR (S1) and GPSGLLGPK (S2) on IEBF of TNF-α stimulated Caco-2 cell monolayers were revealed in Figure 5. Glutamine (Gln) and Arginine (Arg), two nutritious supplements applied in both clinical enteral nutrition and daily dietary supplement to ameliorate intestinal mucosa function, were used here as two positive contrasts.

It is obvious that both S1 and S2 presented significant IEBF protective activity, with a TEER recovery rate of 67.1% and 53.3% (Figure 5A, *p* < 0.05 vs. TNF-α group) and a FD-4 permeability inhibitory rate of 76.6% and 67.9% (Figure 5B, *p* < 0.05 vs. TNF-α group). S1 showed significantly better activity on TEER amelioration compared with Gln and Arg (*p* < 0.05), and on FD-4 permeability compared with Arg (*p* < 0.05). S2 also demonstrated a higher limitation of FD-4 permeability than Arg (*p* < 0.05). The protective effect of identified collagen peptide sequences S1 and S2 on IEBF were comparable or even better, as compared with Gln and Arg in this study.

The physical and chemical properties of peptides are dependent on their constituent amino acids [40]. Based on extensive studies, Gln and Arg have been defined and applied as functional amino acids holds great promise in protecting intestinal mucosa barrier function through regulating IEBF, oxidation or intestinal inflammation under various stress conditions [41,42,43]. Glycine, the most abundant amino acid detected in identified collagen peptides, has been indicated to enhance intestinal mucosal barrier, inhibit oxidative stress and inflammatory responses via regulating tight junction protein, suppressing the activation of NF-*κ*B and the production of inflammatory [44,45]. Proline, a typical amino acid rich in collagen peptides, has been reported to upregulate the tight junction proteins in intestinal epithelial cells [45,46]. Serine, shown in both S1 and S2, has recently been proven to prevent the LPS-induced intestinal barrier dysfunction, inflammatory response, and oxidative stress both in vivo and in vitro [47]. Leucine, one of the major amino acid in S2, can help boost intestinal immune defense system through improving morphological integrity and immunoglobulin production [48,49]. At present, the knowledge about lysine in gut function is limited. Nevertheless, studies with animals proposed that metabolism of lysine is needed to maintain the integrity and function of the gut, as well as to synthesize intestinal mucins and immunoglobulins [50].

It could be reasonably speculated that duo to the combination of these beneficial amino acids, collagen peptides demonstrated more efficient activity compared to Gln and Arg in diminishing TNF-α-induced barrier dysfunction in Caco-2 cell model. Furthermore, compared to free amino acids, the absorption of di- and tri-peptides are more effective in the small intestine, partly contributing to the favorable effect of collagen peptides [51].

## 3. Materials and Methods

### 3.1. Materials

Alaska pollock skin-derived collagen was obtained from Dongyi Tech Co., Ltd (Qingdao, China). Caco-2 cells (HTB-37) were purchased from ATCC (Rockville, MD, USA). All cell culture reagents and supplies were purchased from Thermo Fisher Scientific Co., Ltd. (Shanghai, China). Alcalase, flavourzyme, papain, and trypsin were purchased from Beijing Solarbio Science and Technology Co., Ltd., (Beijing, China). TNF-α, fluorescein isothiocyanate-conjugated dextran 4 kDa (FD-4), and all other reagents used in this study without attribution were obtained from Sigma-Aldrich Chemical Co. (Shanghai, China).

### 3.2. Preparation of APS-Derived Collagen Peptides

Collagen peptides were prepared according to our previous protocol with slightly optimization [1]. In brief, after pH adjusted to 8.0 by 1 M NaOH, 10 mg/mL of APS-derived collagen dissolved in distilled water was hydrolyzed by porcine trypsin (80 U/mg protein), at 45 °C for 2 h. The hydrolyzation was terminated by heating at 100 °C for 10 min. Subsequently, the supernatant was collected after centrifuging at 8000× *g* for 15 min by Jouan BR4i refrigerated centrifuge (Saint-herblain, France), desalted using Sephadex G15 (1.6 × 100 cm) at a flow rate of 1 mL/min over 120 min using ultrapure water as eluent, lyophilized by Martin Christ Alpha 1–4LD (Osterode, Germany), and stored at −20 °C until use.

### 3.3. Separation of Collagen Peptide Fractions Based on Molecular Weight

APS-derived collagen peptides were fractionated based on their molecular weight by Sephadex G25 (1.6 × 80 cm) at a flow rate of 0.5 mL/min over 480 min using ultrapure water as eluent. Bovine serum albumin (66,000 Da), cytochrome C (12,840 Da), insulin (5734 Da), vitamin B12 (1355 Da), glutathione (307.3 Da), and Gly-Gly-Gly (189.2 Da) were used as the molecular weight standards. The logarithm of the standard molecular weights was linearly associated with their respective retention time. All standards and collagen peptides were prepared with ultrapure water, passed through a 0.22 μm low protein-binding membrane filter before being applied to Sephadex G25 column, and then measured at 220 nm. Collagen peptides elution was collected every 5 min. Finally, four fractions were pooled according to their molecular weight (M1, >1500 Da; M2, 1000–1500 Da; M3, 500–1000 Da; M4, <500 Da) and lyophilized for IEBF protective effect assessment. The amount of each collagen peptide fraction was weighed by precision electronic balance.

### 3.4. Separation of Collagen Peptide Fractions Based on Charge Property

The fraction with the highest IEBF protective effect was dissolved in 0.02 M acetate buffer (pH 4.0) and applied to a cation exchange column IexCap SP 6FF (Smart Lifesciences Co., Ltd., Changzhou, Jiangsu, China) using AKTA Explorer (GE Healthcare, Shanghai, China) for separation. The column was equilibrated and first eluted with 0.02 M acetate buffer (pH 4.0) for 60 min; it was subsequently eluted with a concentration of 0–1.0 M NaCl in 0.02 M acetate buffer (pH 4.0) from 60 to 120 min. The flow rate was 0.5 mL/min. Elution was monitored at 220 nm and collected every 2 min. Four major peaks were pooled as E1 to E4 and desalted by Sephadex G15 (1.6 × 100 cm) before being lyophilized for IEBF protective effect evaluation. The amount of each collagen peptide fraction was weighed by precision electronic balance.

### 3.5. Separation of Collagen Peptide Fractions Based on Hydrophobicity

The selected fraction from the last step was further fractionated using an Agilent HPLC system (Agilent Technologies, Inc., CA, USA) equipped with a semi-preparatory Zorbax SB-C18 column (9.4 × 250 mm) and the UV detector set at 220 nm. The mobile phase consisted of solvent A (0.1% trifluoroacetic acid in ultrapure water, v/v) and solvent B (0.1% trifluoroacetic acid in acetonitrile, v/v). Peptides were eluted at room temperature using an isocratic gradient of 10% to 80% solvent B at a flow rate of 0.5 mL/min over 45 min. Elution peaks were collected every minute and four major peaks were pooled and lyophilized as P1 to P4 for IEBF protective effect evaluation. The amount of each collagen peptide fraction was weighed by precision electronic balance.

### 3.6. Identification of High-Active IEBF Protective Collagen Peptide Sequences

The most effective collagen peptide sequences on protecting IEBF were characterized using a Shimadzu 30A UPLC system (Shimadzu Co., Kyoto, Japan) coupled to an ABSCIEX Triple TOFTM 5600+ mass spectrometer (AB Sciex, Foster City, CA, USA) equipped with a DuoSpray ion source following the previous method [2].

Briefly, the UPLC separation was performed on an Acquity UPLC Peptide CSHTM C18 column (2.1 × 100 mm, 1.7 μm) (Water Co., Milford, MA, USA) at a flow rate of 0.25 mL/min at 40 °C. The injection volume was 40 µL. The mobile phase consisted of solvent A (0.1% formic acid in acetonitrile, v/v) and solvent B (0.1% formic acid in water, v/v). The optimized UPLC elution program was as follows: 0–2 min, 95% B; 2–27 min, 95–80% B; 27–30 min, 80–65% B; 37–39 min, 65–20% B; 39–42 min; 20% B; 42–46 min, 95% B.

The mass spectrometry acquisition was operated in positive electrospray ionization (ESI) mode using information-dependent acquisition (IDA) Product Ion. Nitrogen was used as nebulizer and auxiliary gas. The acquisition parameters were as follows: Ion spray voltage, 5500 V; ion source temperature, 550 °C; declustering potential (DP), 100 V; collision energy (CE), 5 V; ion release delay, 67 V; ion release width, 25 V; nebulizer gas pressure, 60 psi; auxiliary gas pressure, 50 psi; curtain gas pressure, 20 psi; Product Ion collection accumulation time, 50 ms. IDA triggering conditions includes: Ions charge between 2–4; ions that exceeded 150 cps; and dynamic background subtract activated. The mass range was set as m/z 100–2000 Da for both TOF MS and TOF MS/MS. The mass spectra were acquired in high-resolution mode with 200 ms and 80 ms accumulation time for TOF MS and TOF MS/MS, respectively.

All MS/MS spectra were collected and processed with Analyst^®^TF Software 1.7 (SCIEX, MA, USA) and then searched against the UniProt SwissProt protein database on ProteinPilot Software 5.0 (SCIEX, MA, USA). Peptide sequence identification was only accepted if the peptide sequence presented an average local confidence above 99%, homogenous (or close homogenous) to fish collagen. The identified peptide sequences were synthesized using a solid-phase method (purity > 99% by HPLC) by China Peptides Co., Ltd (Shanghai, China).

### 3.7. Protection of Collagen Peptides on IEBF

#### 3.7.1. Caco-2 Cell Model

Caco-2 cells from passage 30 to 40 were seeded onto a 12 mm Transwell^®^ insert at a density of 10^5^ cells/cm^2^ and cultivated at 37 °C and humidified 5% CO_2_ atmosphere in the advanced Dulbecco’s modified Eagle’s medium (DMEM) containing 10% (v/v) of fetal bovine serum (FBS), 1% of L-glutamine, 1% of HEPES, and 1% of penicillin-streptomycin for 21 d to simulate the human intestinal epithelial monolayer. During this period, changes in cellular morphology were visualized using a light microscope (CKX31, Olympus Crop., Tokyo, Japan) equipped with a digital camera (SP-350, Olympus Crop., Tokyo, Japan), and increases in trans-epithelial electrical resistance (TEER) were regularly monitored using a Millicell-ERS volt-ohmmeter (Millipore, Bedford, MA). Both apical and basolateral sides of the Caco-2 cell monolayer were rinsed and then bathed with pre-warmed PBS for TEER measurement. Only the monolayers with TEER above 400 Ω·cm^2^ were used for experiments.

#### 3.7.2. The Impact of Collagen Peptides on Barrier Function of Caco-2 Cell Monolayer

The impact of collagen peptides on IEBF was evaluated according to our previous protocol with slight differences [1]. In brief, Caco-2 cell monolayers were designated into different groups as control group, TNF-α group and collagen peptides test groups. After serum deprived overnight, cells in TNF-α group and collagen peptides test groups were treated with 10 ng/mL TNF-α in the basolateral compartment and FBS free medium in the apical compartment for 6 h. In the meantime, control group was maintained in FBS free medium in both apical and basolateral sides. Subsequently, the apical compartment medium of collagen peptides test groups was replaced with freshly prepared 1 mg/mL of collagen peptides fractions correspondingly in FBS free medium and those of control and TNF-α group were replaced with only FBS free medium as control group. After being incubated for another 24 h, TEER and the permeability of 4 kDa fluorescein isothiocyanate-conjugated dextran (4 kDa FITC-dextran, FD-4) were measured to show the IEBF protective effect of collagen peptides. All tests were performed with nine repeats.

#### 3.7.3. FD-4 Permeability

FD-4 permeability was determined as described previously [3]. Briefly, monolayers were gently washed with pre-warmed Hank’s balanced salt solution (HBSS). Medium in the apical compartment was gently aspirated and replaced with 1 mg/mL FD-4 in HBSS; medium in basolateral compartment was replaced with only HBSS. After incubation at 37 °C and 5% CO_2_ atmosphere for 2 h, 100 μL basolateral medium was collected for the measurement of fluorescence at Ex 480/Em 520 nm. 

### 3.8. Statistics

Statistical analysis was performed by Prism 7, version 7.0a (GraphPad Software Inc., San Diego, CA, USA). All values are presented as mean ± SD. Differences among groups were analyzed using the Tukey’s multiple comparisons test. Statistical significance was accepted when values of *p* < 0.05.

## 4. Conclusions

In this study, innovative research on identification and structure–activity relationship of collagen peptide in IEBF protection has been done. The results substantiate that the lower molecular weight (500–1000 Da) and higher hydrophilicity may be the features of high-efficiency IEBF protective collagen peptides. Two sequences, GPSGPQGSR and GPSGLLGPK, were identified from the high-efficiency IEBF protective collagen peptide fraction, which could be used for further studies about the action route and mechanism of IEBF protective peptides on molecular biology level, and for the development of functional food and pharmaceuticals.

## Figures and Tables

**Table 1:** Sequences identified from high intestinal epithelial barrier function (IEBF) protective Alaska pollock collagen peptide fraction P1.

Peptide Sequences	Molecular Weight (Da)	Homogenous (or Close Homogenous) to:	
		Organism	Protein
GPSGPQGSR	841.4098511	Gasterosteus aculeatus	Collagen alpha-2(I) chain
GPSGLLGPK	824.3828735	Ictalurus punctatus	Collagen alpha-1(XXI) chain
